# Cloning and Expression of *Aspergillus tamarii* FS132 Lipase Gene in *Pichia pastoris*

**DOI:** 10.3390/ijms11062373

**Published:** 2010-06-03

**Authors:** Bihong Shi, Liqing Zeng, Haolei Song, Qiaoqin Shi, Songgang Wu

**Affiliations:** 1 College of Life Sciences, Fujian Normal University, The University Town, Min-hou, Fuzhou 350108, China; E-Mails: zenglq_mail@163.com (L.Z.); songhl_mail@163.com (H.S.); 2 Engineering Research Center of Industrial Microbiology, Ministry of Education, Fujian Normal University, Fuzhou 350108, China; E-Mails: sqq@fjnu.edu.cn (Q.S.); wsg5014242@yahoo.com.cn (S.W.)

**Keywords:** *Aspergillus tamarii*, lipase, gene cloning, expression, *Pichia pastoris*

## Abstract

A lipase gene (*atl*) was cloned from *Aspergillus tamarii* FS132 for the first time. The gene was found to have an open reading frame of 1024 base pairs (bp), and the coding region of the gene contained two introns (51 bp and 52 bp). Multi-alignment analysis of the deduced amino acid sequence indicated high homology between the enzyme and mono-and diacylglycerol lipases from fungi *Aspergillus*. The recombinant lipase was expressed in *Pichia pastoris* GS115 cells. The recombinant lipase was found to have a molecular mass of 36.7 kDa, and it exhibited lipase activity of 20 U/mL in culture supernatant when tributyrin was used as the substrate.

## 1. Introduction

Lipases (triacylglycerol acylhydrolases, EC3.1.1.3) act at fat-water interfaces to hydrolyze long-chain triglycerides to fatty acids and glycerol or to catalyze the reaction reversely under certain conditions [1]. They are widely used in the dairy and oleo chemical industries, in the synthesis of structured triglycerides, surfactants, pharmaceuticals and agrochemicals, and in polymers and household detergents [2]. Lipases of microbial origin represent the most extensively used class and are receiving increasing attention due to their relative ease of production and potential applications in biotechnology. This interest stems from their ability to hydrolyze ester bonds, trans-esterify triglycerides and resolve racemic mixtures as well as their ability to synthesize ester and peptide bonds in the reverse mode [2].

Many microbial lipase genes have been cloned during past few decades. Several of these genes are from fungi, including *Aspergillus oryzae* [3–5], *Rhizopus delemar* [6], *Rhizomucor miehei* [7], *Penicillium camembertii* [8], *Penicillium cyclopium* [9], and *Geotrichum candidum* [10]. Most of these lipases have been expressed heterogeneously and their properties characterized [11–14]. The filamentous fungi *Aspergillus tamarii* was also found to produce a lipase, and the enzymological characteristics of its lipase was reported in 1995 [15]. However, the DNA sequence of the lipase from *Aspergillus tamarii* is not currently available.

Once a desired gene has been cloned, it is often heterologously expressed and its function characterized. *Pichia pastoris* has proven to be highly popular as a host for overexpression of eukaryotic heterologous proteins [16]. To date, many microbial lipase genes have been cloned and expressed in *P. pastoris* [17–19].

In this article, we report the first cloning and sequencing of the genomic DNA and cDNA of the lipase gene from *Aspergillus tamarii* FS132 (ATL) and its expression in *P. pastoris* GS115, so as to characterize and further determine the function of this lipase.

## 2. Results and Discussion

### 2.1. *Cloning of the* Aspergillus tamarii *FS132 Lipase Genomic DNA and cDNA*

PCR and RT-PCR amplicons of genomic DNA and cDNA, respectively, of the lipase encoding gene from *Aspergillus tamarii* FS132 (ATL), were cloned. The amplified products were purified and inserted into the pMD19-T vector. Then, the recombinant plasmids pMD19-T/ATL and pMD19-T/ATL-cDNA were transformed into *E. coli* JM109 cells. The target DNA of ATL-DNA and ATL-cDNA were confirmed by sequence determination.

The sequences of the cloned genomic DNA and cDNA of the ATL lipase were deposited in GenBank under the accession numbers of EF198417 and EU131679, respectively.

### 2.2. Sequence Analysis

The cloned genomic DNA sequence was aligned with *Aspergillus* lipase gene sequences in the GeneBank database using BLAST. The total length of the cloned genomic DNA was 1742 base pairs (bp), which included 378 bp of 5′ non-coding region, 340 bp of 3′ non-coding region, and 1024 bp of open reading frame (ORF) between bp 379 and 1402 (accession no. EF198417). The GC content of the gene was 50.68%.

**Figure 1:**
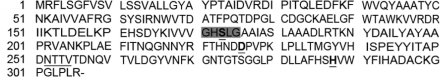
The deduced ATL amino acid sequence. The conserved sequence G-X-S-X-G in common lipases is indicated by the shadowed box, the catalytic triad Ser173-Asp 226-His 288 is in boldfont and underlined, and the putative glycosylation site 252–255, NTTV, is underlined.

The cloned cDNA was 921 bp in size (accession no. EU131679). The deduced amino acid sequence consisted of 306 amino acid residues, with a predicted molecular mass of 33.456 kDa and pI of 5.46. The conserved pentapeptide Gly-X-Ser-X-Gly in most microbial lipases was also found in the predictive ATL (Figure 1). This peptide is thought to play an essential role in substrate recognition and binding [20]. The three amino acids (serine, aspartic acid and histidine) of the catalytic triad found in most lipases were also present in the deduced amino acid sequence. Their location in the sequence is shown as Ser (173)-Asp (226)-His (288).

Comparison of the putative ATL-DNA and ATL-cDNA sequences showed a coding region of 1024 bp in the predicted ATL-DNA with two introns sized 51 bp and 52 bp. The introns were found at 86–136 bp and 306–357 bp in the ATL-DNA sequence, respectively (assume the first base A of the initial codon ATG as the first site of the ATL gene). The size of the introns was similar to introns of other lipase genes from the same genus of *Aspergillus* [4,21].

The homology comparison revealed that the predicted ATL gene showed high similarity with lipases from *Aspergillus oryzae* (99%, D85895), *Aspergillus flavus* (99%, AF404489) and *Aspergillus parasiticus* (94%, AF404488) at the DNA level.

**Figure 2:**
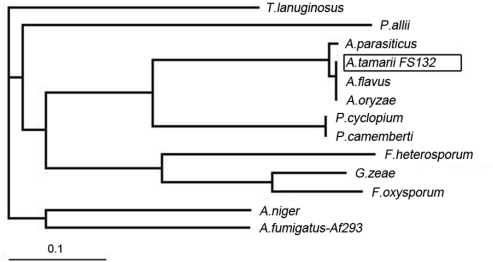
Phylogenetic analysis of the lipase gene from *Aspergillus tamarii* FS132. The dendrogram shows the sequence relationships between *Aspergillus tamarii* FS132 (ABM90643) and *Thermomyces lanuginosus* (O59952), *Penicillium allii* (AAP59844), *Aspergillus parasiticus* (AAO17920), *Aspergillus flavus* (AAO17921), *Aspergillus oryzae* (BAA12912), *Penicillium cyclopium* (AAF99710), *Penicillium camemberti* (BAA14345), *Fusarium heterosporum* (AAB34680), *Gibberella zeae* (AAQ23181), *Fusarium oxysporum* (ABR12479), *Aspergillus niger* (ABG37906), and *Aspergillus fumigatus*-Af293 (XP_748138).

The deduced ATL amino acid sequence was multi-aligned with twelve fungal lipases obtained from GenBank and showed high similarity to other lipases in the predicted active-site regions. However, the overall similarities varied largely between the different lipases compared. The deduced ATL amino acid sequence showed exact identity with the characterized genes *lip*A from *Aspergillus flavus* (AAO17921) and *mdl* B from *Aspergillus oryzae* (BAA12912), 98% identity with a lipase from *A. parasiticus* (AAO17920), 62% identity with lipases from *P. cyclopium* (AAF99710) and *P. camemberti* (BAA14345), 42% similarity with a lipase from *A. fumigatus* (XP_748138), and lower similarity (<40%) with lipases from the remaining fungi (Figure 2). A phylogenetic tree was constructed by the neighbor joining method to reveal the relationship between the different lipases analyzed (Figure 2). The lipases from *A. tamarii* FS132, *A. flavus, A. oryzae* and *A. parasiticus* were placed in the same sub-group based on their high sequence homology with each other. The high similarity between the ATL and the identified lipase of *A. oryzae* and *A. flavus* showed that the cloned lipase belonged to the mono and diacylglycerol lipase family from filamentous fungi [4,21]. The high similarity of theses lipase genes is probably related to the high phylogenic homology of their respective hosts based on the 18S rRNA [22].

### 2.3. *Expression of ATL in* P. pastoris *GS115*

**Figure 3:**
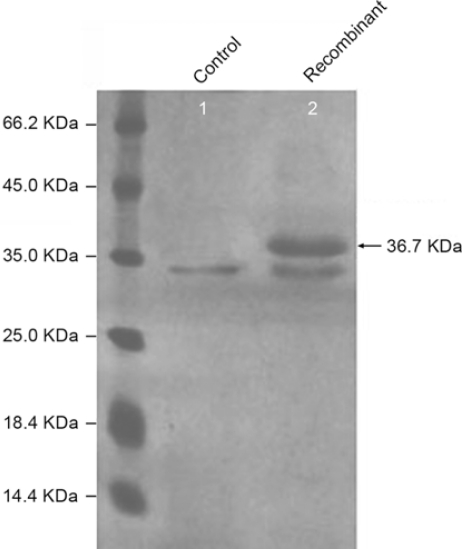
SDS-PAGE analysis of the expressed protein. Lane M, protein molecular marker. Lane 1, culture supernatant of *Pichia pastoris* GS115 transformed with the empty plasmid pPIC3.5K induced as negative control. Lane 2, culture supernatant of recombinant *Pichia pastoris* GS115-pPIC3.5K/ATL-cDNA after induction by methanol. The band corresponding to recombinant lipase is shown by the arrow.

The *P. pastoris* GS115 cells harboring the pPIC3.5K/ATL-cDNA were induced with methanol to a final concentration of 0.5% and incubated as described in the Experimental section. The supernatants of the cell cultures revealed the presence of a new protein with an approximate molecular weight of 36.7 kDa, as measured by SDS-PAGE, which was not observed in the cell-free supernatant of host *P. pastoris* GS115 cells with the empty vector pPIC3.5K (Figure 3).

According to the predicted amino acids sequence of ATL, a putative signal peptide was present in the 306 deduced amino acids between Met1 and Arg28 (Figure 1), which shows features of signal peptides with a basic amino acid (Arg) at position 2 followed by a long hydrophobic region [23]. The putative mature lipase of ATL consisted of 278 amino acids with a calculated molecular mass of 30.4 kDa following removal of 28 amino acids of signal peptides. This size differed from the apparent size determined by SDS-PAGE (36.7 kDa, Figure 3). Similar findings were reported for lipases from *A. oryzae* [4] and *P. camembertii* [8]. The discrepancy in molecular weight between the native expressed mature lipase and the deduced molecular mass was probably due to glycosylation of the enzyme. One potential N-glycosylation site [4], NTTV, was also found in the ATL amino acid sequences at positions 252–255 (Figure 1).

Qualitative measurement of ATL activity in the cell-free culture showed the formation of a clear zone on TMM agar plates. In contrast, no clear zone developed for the negative sample on the same plate (data not show). The crude enzyme in the supernatant exhibited 20 U/mL of lipase activity when tributyrin was used as a substrate, while no clear zone formed on the OMM plate for the same sample. This finding may be related to the low lipase activity of the recombinant enzyme (20 U/mL of lipase activity). A similar result was found for the native lipase of *A. tamarii* FS132 [24]. Further optimization of the culture strategies is necessary for improving the enzyme activity [19,25]. Many studies demonstrated that the lipase productivity can be increased remarkably after optimizing the culture strategies of *P. pastoris* [19,25]. Minning *et al.* showed that the *Rhizopus oryzae* lipase had 2.5-fold higher productivity compared to the initial cultivation after altering the feeding strategy of methanol to the culture medium of *P. pastoris* [25]. The optimization of *A. tamarii* FS132 lipase production in *P. pastoris* is currently ongoing.

## 3. Experimental Section

### 3.1. Strains, Plasmids and Culture Medium

*Aspergillus tamarii* FS132, isolated from the volcanic vent soil in Xinjiang Uygur Autonomous Region, China, was grown as described previously [24] and used as the source of genomic DNA and total RNA. The plasmid pMD19-T (TaKaRa, Dalian, China) was used for TA cloning of the genomic DNA and cDNA of the lipase gene. The structure map of the pMD19-T vector is available online (www.takara.com.cn). pBluescript II KS (+) (Stratagene) and pPIC3.5K (Invitrogen) were used for vector construction, propagation and expression of the lipase gene. The *E. coli* JM109 strain and the *Pichia pastoris* GS115 strain were used for lipase gene cloning and expression, respectively.

The *E. coli* JM109 strain was cultivated in Luria-Bertani (LB) medium. The recombinant *E. coli* colonies were selected from the LB agar plates containing 100 μg/L ampicillin at 37 °C. *P. pastoris* GS115 was cultivated in yeast extract peptone dextrose (YPD) medium (1% yeast extract, 2% peptone and 2% of dextrose). Minimal dextrose (MD) medium (1.34% yeast nitrogen base [YNB], 4 × 10^−5^% biotin, 2% dextrose) plates were used to screen His^+^ yeast transformants. Minimal methanol (MM) medium (1.34% YNB, 4 × 10^−5^% biotin, and 1% methanol) coupled with MD plates were used to identify the phenotypes Mut^+^ (methanol utilize normally) and Mut^s^ (methanol utilize slowly) of recombinants [26]. The MM medium supplemented with 2% tributyrin (TMM) or 2% olive oil (OMM) was used for screening lipase expressing recombinants. The recombinant *P. pastoris* was grown on buffered glycerol complex (BMGY) medium (1% yeast extract, 2% peptone, 1.34% YNB, 4 × 10^−5^% biotin, 1% glycerol, 100 mM potassium phosphate buffer pH 6.0) and lipase expression was induced in buffered methanol complex (BMMY) medium (1% yeast extract, 2% peptone, 1.34% YNB, 4 × 10^−5^% biotin, 0.5% methanol, 100 mM potassium phosphate buffer, pH 6.0) at 30 °C.

### 3.2. *Cloning of the* Aspergillus tamarii *FS132 Lipase Gene (Atl)*

The genomic DNA of *Aspergillus tamarii* FS132 was isolated using the salt extraction method [27]. The *atl* gene was amplified from the genomic DNA by PCR using forward primer F1 (5′-CCAAGCTTTGCAACCAAGCCTGTCG-3′) and reverse primer R1 (5′-CTGCAGGTGTAGTGTGCTTGGCCGA-3′) [21]. The PCR reaction proceeded by an initial denaturing step for 2 min at 95 °C followed by 30 cycles of denaturing at 94 °C for 15 s, annealing at 56 °C for 30 s, and extension at 72 °C for 2 min. The reaction was terminated by a final extension at 72 °C for 10 min. The PCR product was detected on a 1% (w/v) agarose gel and purified with a DNA purification kit (TIANGEN, Beijing).

The PCR fragment was ligated into the pMD19-T vector and transformed into *E. coli* JM109 competent cells using a standard protocol [28]. Positive recombinant plasmids were selected by the white/blue selection method and identified by colony PCR. The plasmid-ATL DNA was extracted and purified from *E. coli* JM109 cells. The ATL DNA was then sequenced by Takara Co. Ltd, and the DNA sequence of the *atl* gene was submitted to GenBank.

The total RNA of *Aspergillus tamarii* FS132 was isolated using the Trizol agent (Invitrogen) according to the instructions provided by the manufacturer. The first chain of cDNA was synthesized by the SMART™ RACE cDNA Amplification Kit (Clontech). For RT-PCR, the forward primer F2 (5′-TTAGCGCAATGGCAATCCAGGACCC-3′) and reverse primer R2 (5′-TTAGCGCAATGGCAATCCAGGACCC-3′) were designed according to conserved sequences at both ends of the lipase coding region from *Aspergillus* genus in GenBank. The RT-PCR was performed by an initial denaturing step of 4 min at 94 °C followed by 30 cycles of denaturing at 94 °C for 45 s, annealing at 60 °C for 30 s, and extension at 72 °C for 1.5 min. The reaction was terminated by a final extension at 72 °C for 10 min. The PCR product was examined on a 1% (w/v) agarose gel and purified by a DNA purification kit (TIANGEN, Beijing).

The purified product was ligated into the pMD19-T vector and transformed into *E. coli* JM109. The positive recombinant colonies were selected and identified, the recombinant plasmid (pMD19-T/ATL-cDNA) was purified from *E. coli* JM109 cells and the ATL-cDNA was sequenced by Takara Co. Ltd. The cDNA sequence of the *atl* gene was submitted to GenBank.

### 3.3. Sequence Analysis

Sequence similarity searches were performed in the GenBank Database using the BLAST program. The sequences were multiple aligned by the program Clustal X1.83. A phylogenetic dendrogram based on the aligned sequences were produced using the software Treeview (http://taxonomy.zoology.gla.ac.uk/rod/treeview.html) according to the neighboring-joining method [29].

### 3.4. Construction of the Expression System

The recombinant vector pMD19-T/ATL-cDNA was digested with *Pst*I and *Bam*HI. The ATL-cDNA was ligated into the pBluescript ks (+) vector (abbreviate as pBks in the following) and transformed into *E. coli* JM109 cells. The pBks /ATL-cDNA plasmid DNA was extracted and double digested with *Eco*RI and *Bam*HI and detected by agarose gel. The target DNA was purified and ligated into the plasmid pPIC3.5K, which was double digested with the same two endonucleases. The detail protocols were referred by J. Sambrook [28].

The recombinant expression vector pPIC3.5K/ATL-cDNA was linearized by *Sac*I and electroporated into *P. pastoris* GS115 competent cells via pulse discharge under conditions of 750 V, 25 μF for 4 ms (Bio-Rad Gene Pulser). The His^+^ yeast transformants were initially selected on MD agar plates for their capacity to grow in the absence of histidine and directly screened for clones with phenotypes of Mut^+^ and lipase expression on TMM and OMM plates. The positive clones with lipase activity, which formed the transparent zone on TMM or OMM plates, were selected and verified by PCR. The forward primer F3 (5′-GACTGGTTCCAATTGACAAGC-3′) and reverse primer R3 (5′-GCAAATGGCATTCTGACATCC-3′) for PCR were designed according to the sequences for homologous recombination (AOX regions) in the pPIC3.5K plasmid. The PCR was performed by an initial denaturing step of 2 min at 94 °C followed by 30 cycles of 94 °C for 1 min, 54 °C for 1 min, and 72 °C for 1.5 min. The reaction was terminated at 72 °C for 7 min. The amplifications were detected by 1% of agarose gels.

### 3.5. Induction of Atl Gene Expression in Yeast

The positive transformants of *P. pastoris* GS115 were grown in the BMGY media with vigorous shaking at 30 °C until the OD_600_ value reached 2–6. Cells were harvested and re-suspended in the BMMY media to an OD_600_ of 1.0. The *atl* expression was induced by continuous incubation at 30 °C for 96 hours and the addition of methanol every 24 hours to a final concentration of 0.5%. The supernatant of the induced culture was used for the lipase activity assay and expression protein identification. A negative control containing the empty pPIC3.5K vector was carried out in parallel.

### 3.6. Identification of the Expression Product

Lipase expressing recombinants were screened on TMM agar plate [30]. Clear zones were formed by adding lipase-producing supernatant culture to TMM agar plate. SDS-PAGE was used to examine the expression level of the target protein and evaluate the molecular mass of the protein. Lipase activity was determined by the titration method, with tributyrin as the substrate emulsion. One milliliter of diluted supernatant was added to 5 mL of tributyrin emulsion and 4 mL of 0.05 M glycine-sodium hydroxide buffer (pH 9.4). Samples were incubated at 37 °C for 10 min with gentle shaking. The reaction was stopped by addition 20 mL of 100% ethanol containing 0.1% phenolphthalein indicator. Lipase activity was determined by titration of the released fatty acid with 0.05 M sodium hydroxide. One unit of lipase activity was defined as the amount of enzyme that liberated 1 μmol of fatty acid per minute.

## 4. Conclusions

*Aspergillus* lipase is one of an important class of industrial lipases. In this work, we first cloned a lipase gene from *Aspergillus tamarii* FS132. The total length of the cloned lipase genomic DNA was 1742 bp, which contained 378 bp of 5′ non-coding region, 340 bp of 3′ non-coding region, and 1024 bp of open reading frame (ORF) (accession no. EF198417). The cloned cDNA was 921 bp in size (accession no. EU131679). The lipase gene encodes a 306 amino acid protein, which contains the conserved motif Gly-X-Ser-X-Gly present in most microbial lipases. Phylogenetic analysis suggested that the cloned lipase belongs to the mono- and diacylglycerol lipases from fungi *Aspergillus*.

The function of the cloned gene was further characterized in *P. pastoris* GS115. The recombinant lipase was expressed in GS115 cells and showed lipase activity of 20 U/mL with the tributyrin as the substrate. The recombinant lipase exhibited a molecular mass of 36.7 kDa in SDS-PAGE gel, which is larger than the predicted molecular mass (30.4 kDa for the mature lipase). The discrepancy may be due to glycosylation of the enzyme.

## References

[1] Jaeger KE Eggert T Lipases for biotechnology Curr. Opin. Biotechnol 2002 13 390 397 12323363 10.1016/s0958-1669(02)00341-5

[2] Hasan F Shah AA Hameed A Industrial applications of microbial lipases Enzyme Microb. Technol 2006 39 235 251

[3] Ohnishi K Toida J Nakazawa H Sekiguchi J Genome structure and nucleotide sequence of a lipolytic enzyme gene of *Aspergillus oryzae* FEMS Microbiol. Lett 1995 126 145 150 7705606 10.1111/j.1574-6968.1995.tb07408.x

[4] Tsuchiya A Nakazawa H Toida J Ohnishi K Sekiguchi J Cloning and nucleotide sequence of the mono- and diacylglycerol lipase gene (mdlB) of *Aspergillus oryzae* FEMS Microbiol. Lett 1996 143 63 67 8807803 10.1111/j.1574-6968.1996.tb08462.x

[5] Toida J Fukuzawa M Kobayashi G Ito K Sekiguchi J Cloning and sequencing of the triacylglycerol lipase gene of *Aspergillus oryzae* and its expression in *Escherichia coli* FEMS Microbiol. Lett 2000 189 159 164 10930731 10.1111/j.1574-6968.2000.tb09223.x

[6] Haas MJ Allen J Berka TR Cloning, expression and characterization of a cDNA encoding a lipase from *Rhizopus delemar* Gene 1991 109 107 113 1756969 10.1016/0378-1119(91)90594-2

[7] Boel E Huge-Jensen B Christensen M Thim L Fiil NP *Rhizomucor miehei* triglyceride lipase is synthesized as a precursor Lipids 1988 23 701 706 3419283 10.1007/BF02535672

[8] Yamaguchi S Mase T Takeuchi K Cloning and structure of the mono- and diacylglycerol lipase-encoding gene from *Penicillium camembertii* U-150 Gene 1991 103 61 67 1879699 10.1016/0378-1119(91)90391-n

[9] Wu M Qian Z Jiang P Min T Sun C Huang W Cloning of an alkaline lipase gene from *Penicillium cyclopium* and its expression in *Escherichia coli* Lipids 2003 38 191 199 12784858 10.1007/s11745-003-1051-7

[10] Shimada Y Sugihara A Tominaga Y Iizumi T Tsunasawa S cDNA molecular cloning of *Geotrichum candidum* lipase J. Biochem 1989 106 383 388 2481674 10.1093/oxfordjournals.jbchem.a122862

[11] Jaeger KE Eggert T LipasIsobe K Nokihara K Yamaguchi S Mase T Schmid RD Crystallization and characterization of monoacylglycerol and diacylglycerol lipase from *Penicillium camembertii* Eur. J. Biochem 1992 203 233 237 1730229 10.1111/j.1432-1033.1992.tb19851.x

[12] Vernet T Ziomek E Recktenwald A Schrag JD de Montigny C Tessier DC Thomas DY Cygler M Cloning and expression of *Geotrichum candidum* lipase II gene in yeast. Probing of the enzyme active site by site-directed mutagenesis J. Biol. Chem 1993 268 26212 26219 7902836

[13] Isobe K Nokihara K Primary structure determination of mono- and diacylglycerol lipase from *Penicillium camembertii* FEBS Lett 1993 320 101 106 8458423 10.1016/0014-5793(93)80071-2

[14] Jeon JH Kim JT Kim YJ Kim HK Lee HS Kang SG Kim SJ Lee JH Cloning and characterization of a new cold-active lipase from a deep-sea sediment metagenome Appl. Microbiol. Biotechnol 2009 81 865 874 18773201 10.1007/s00253-008-1656-2

[15] Saad RR Production of lipase from *Aspergillus tamarii* and its compatability with commercial detergents-culture medium and formation conditions optimization for enzyme production for use in Surfacatant composition Folia Microbiol 1995 40 263 266

[16] Cereghino JL Cregg JM Heterologous protein expression in the methylotrophic yeast *Pichia pastoris* FEMS Microbiol. Rev 2000 24 45 66 10640598 10.1111/j.1574-6976.2000.tb00532.x

[17] Holmquist M Tessier DC Cygler M High-level production of recombinant *Geotrichum candidum* lipases in yeast *Pichia pastoris* Protein Expr. Purif 1997 11 35 40 9325136 10.1006/prep.1997.0747

[18] Resina D Serrano A Valero F Ferrer P Expression of a *Rhizopus oryzae* lipase in *Pichia pastoris* under control of the nitrogen source-regulated formaldehyde dehydrogenase promoter J. Biotechnol 2004 109 103 113 15063618 10.1016/j.jbiotec.2003.10.029

[19] Surribas A Stahn R Montesinos JL Enfors SO Valero F Jahic M Production of a *Rhizopus oryzae* lipase from *Pichia pastoris* using alternative operational strategies J. Biotechnol 2007 130 291 299 17544535 10.1016/j.jbiotec.2007.04.009

[20] Tan Y Miller KJ Cloning, expression, and nucleotide sequence of a lipase gene from *Pseudomonas fluorescens* B52 Appl. Environ. Microbiol 1992 58 1402 1407 1599260 10.1128/aem.58.4.1402-1407.1992 PMC195611

[21] Yu J Mohawed SM Bhatnagar D Cleveland TE Substrate-induced lipase gene expression and aflatoxin production in *Aspergillus parasiticus* and *Aspergillus flavus* J. Appl. Microbiol 2003 95 1334 1342 14633008 10.1046/j.1365-2672.2003.02096.x

[22] Nikkuni S Nakajima H Hoshina SI Ohno M Suzuki C Kashiwagi Y Mori K Evolutionary relationships among *Aspergillus oryzae* and related species based on the sequences of 18S rRNA genes and internal transcribed spacers J. Gen. Appl. Microbiol 1998 44 225 230 12501432 10.2323/jgam.44.225

[23] Perlman D Halvorson HO A putative signal peptidase recognition site and sequence in eukaryotic and prokaryotic signal peptides J. Mol. Biol 1983 167 391 409 6345794 10.1016/s0022-2836(83)80341-6

[24] Zeng L Shi B Zhou X Zeng Q Shi Q Preliminary optimization of the culture conditions for lipase producing strain *Aspergillus sp.* FS132 and comparative analysis of its 18S rRNA gene Ind Microbiol 2007 37 11 15 (in Chinese).

[25] Minning S Serrano A Ferrer P Sola C Schmid RD Valero F Optimization of the high-level production of *Rhizopus oryzae* lipase in *Pichia pastoris* J. Biotechnol 2001 86 59 70 11223145 10.1016/s0168-1656(00)00402-8

[26] Yang J Zhang B Yan Y Cloning and expression of *Pseudomonas fluorescens* 26-2 lipase gene in *Pichia pastoris* and characterizing for transesterification Appl. Biochem. Biotechnol 2009 159 355 365 19005622 10.1007/s12010-008-8419-5

[27] Aljanabi SM Martinez I Universal and rapid salt-extraction of high quality genomic DNA for PCR-based techniques Nucleic Acids Res 1997 25 4692 4693 9358185 10.1093/nar/25.22.4692 PMC147078

[28] Sambrook J Russell DW Molecular Cloning: A Laboratory Manual Cold spring harbor laboratory Press New York, NY, USA 2001

[29] Kimura M A simple method for estimating evolutionary rates of base substitutions through comparative studies of nucleotide sequences J. Mol. Evol 1980 16 111 120 7463489 10.1007/BF01731581

[30] Samad MYA Salleh AB Razak CNA Ampon K Yunus WMZW Basri M A lipase from a newly isolated thermophilic *Rhizopus rhizopodiformis* World J. Microb. Biotechnol 1990 6 390 394 10.1007/BF01202120 24430138

